# Repurposing HIV-Protease Inhibitor Precursors as Anticancer Agents: The Synthetic Molecule RDD-142 Delays Cell Cycle Progression and Induces Autophagy in HepG2 Cells with Enhanced Efficacy via Liposomal Formulation

**DOI:** 10.3390/ijms262110305

**Published:** 2025-10-23

**Authors:** Fabiana Crispo, Antonio Vassallo, Immacolata Faraone, Alessandro Santarsiere, Lucia Chiummiento, Mara Martinelli, Nicoletta Cascelli, Xavier Fernàndez-Busquets, Rocchina Miglionico, Ilaria Nigro, Carla Caddeo, Maria Francesca Armentano

**Affiliations:** 1 Laboratory of Preclinical and Translational Research, IRCCS-CROB, Referral Cancer Center of Basilicata, 85028 Rionero in Vulture, Italy; fabiana.crispo@crob.it (F.C.); mara.martinelli@crob.it (M.M.);; 2 Department of Health Sciences, University of Basilicata, 85100 Potenza, Italy; antonio.vassallo@unibas.it (A.V.); immacolata.faraone@unibas.it (I.F.); mariafrancesca.armentano@unibas.it (M.F.A.); 3 Department of Basic and Applied Science, University of Basilicata, 85100 Potenza, Italy; alessandro.santarsiere@unibas.it (A.S.); lucia.chiummiento@unibas.it (L.C.); 4 Institute for Bioengineering of Catalonia (IBEC), The Barcelona Institute of Science and Technology, Baldiri Reixac 10-12, 08028 Barcelona, Spain; xfernandez_busquets@ub.edu; 5 Barcelona Institute for Global Health (ISGlobal, Hospital Clínic-Universitat de Barcelona), Rosselló 149-153, 08036 Barcelona, Spain; 6 Nanoscience and Nanotechnology Institute (IN2UB), University of Barcelona, Martí i Franquès 1, 08028 Barcelona, Spain; 7 Department of Life and Environmental Sciences, University of Cagliari, S.P. Monserrato-Sestu Km 0.700, 09042 Monserrato, Italy; caddeoc@unica.it

**Keywords:** drug repositioning, HIV-protease inhibitor precursor, liposomes, hepatocellular carcinoma, cell cycle delay, proteasome inhibitors

## Abstract

Hepatocellular carcinoma (HCC) remains a global health issue due to high incidence and mortality, complicated by limited therapeutic options and evolution of de novo resistance to conventional chemotherapy. In this study, we investigated the antiproliferative activity of RDD-142, a synthetic precursor of the HIV-1 protease inhibitor (HIV-PI) Darunavir analog, on the human hepatocellular carcinoma line (HepG2) and healthy hepatocyte line (IHH), both as a free molecule and in liposomal formulation. RDD-142 demonstrated a selective cytostatic effect on HepG2, preserving healthy IHH cells. Mechanistically, RDD-142 delayed cancer cell proliferation by attenuating the ERK1/2 signaling pathway, and concurrently, it activated the autophagic process via p62 up-regulation. These effects were linked to RDD-142 inhibitory activity on the chymotrypsin-like subunit of the proteasome, triggering a UPR-mediated stress response. Notably, the liposomal formulation of RDD-142 significantly enhanced intracellular intake and cytotoxic efficacy. RDD-142 demonstrated promising potential as a therapeutic agent for HCC. Its antitumor activity may be further amplified through liposomal nanoformulation, offering a successful strategy to reduce effective dosage and minimize adverse effects.

## 1. Introduction

Liver cancer, which ranks as the sixth most frequently diagnosed malignancy and the third leading cause of cancer-related deaths worldwide, represents a growing challenge for public health systems. The most common form of primary liver cancer is hepatocellular carcinoma (HCC), which accounts for approximately 80% of all cases [1,2].

The management of patients with HCC requires a multidisciplinary approach and depends on the tumor stage, the extent of liver dysfunction, and the patient’s overall condition. Early-stage HCC may be effectively treated with curative approaches, including surgical resection, local ablation, or liver transplantation. In contrast, advanced-stage HCC is typically managed with systemic therapies, which often remain suboptimal due to the frequent development of drug resistance and high cytotoxicity [3,4]. This highlights the urgent need for innovative treatment approaches with high anti-tumor efficacy and minimal side effects.

Two major challenges currently hinder the development of new anticancer agents. The first concerns the high costs, long timelines, and significant failure rates associated with conventional drug discovery, which have fueled increasing interest in drug repurposing. This strategy, based on the identification of new therapeutic indications for already approved drugs, allows a significant reduction in both development time and cost, as pharmacokinetic, pharmacodynamic, and toxicity profiles are already well-established, and early preclinical studies can be bypassed. Consequently, the average time required for drug approval can be reduced from 10 to 17 years to approximately 3–12 years, at roughly half the cost [5,6]. Notably, about 30% of new oncology clinical trials now include repositioned drugs, highlighting the growing relevance of this approach in cancer research [7,8]

Among the drugs explored within this strategy, human immunodeficiency virus (HIV) protease inhibitors (HIV-PIs), such as Ritonavir, Nelfinavir, and Saquinavir, have shown promising antitumor activity across several tumor types [9,10,11,12]. Proposed mechanisms of action include the induction of apoptosis, autophagy, and endoplasmic reticulum (ER) stress; interference with the cell cycle; induction of oxidative stress and mitochondrial damage; and inhibition of angiogenesis and tumor cell invasion [13,14,15,16,17]. These pleiotropic activities represent a solid basis for the identification of new therapeutic options, responding to the urgent need for drugs capable of overcoming the limitations of currently available therapies, including drug resistance, both intrinsic and acquired, toxicity, low bioavailability, and significant side effects [18].

However, although several studies have investigated HIV-PIs in systemic solid tumors and HIV-associated malignancies, only a few have explored their potential applications in HCC.

The second challenge facing drug developers is related to the intrinsic pharmacokinetic limitations of many therapeutic molecules, including poor solubility, short circulation time, and the need for controlled release. To overcome these barriers, nanoparticle delivery systems have emerged as powerful tools. Among them, liposomes are the very first generation of nanoparticle-based therapy approved for the treatment of cancer, and still are the best-investigated platform in clinical trials and academic research [19,20,21,22]. They offer numerous advantages over therapy with free drugs: increased solubility and bioavailability of the loaded drug, controlled drug release, drug protection, and selectivity against cancer targets. These factors thus increased efficacy and reduced drug dose, which lowers systemic toxicity [23,24,25].

**Figure 1:**
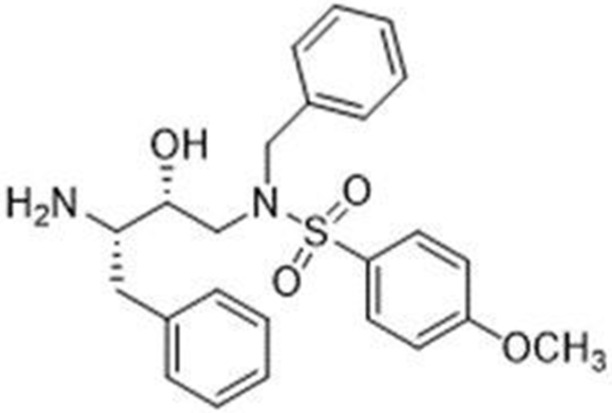
Chemical structure of RDD-142.

Based on these considerations, and to further explore the repositioning potential of HIV-PIs in HCC, we focused our attention on the synthetic compound (*N*-((2*R*,3*S*)-3-amino-2-hydroxy-4-phenylbutyl)-N-benzyl methoxybenzenesulfonamide), called RDD-142 (Figure 1) [26].

The synthetic molecule RDD-142 is a precursor of the analogous commercial HIV-PI Darunavir and has already shown the ability to impair the viability of HepG2 hepatocarcinoma cells through multiple mechanisms of action [27]. In the present study, we investigated its cytostatic effects at lower concentrations and further characterized its molecular mechanisms. Additionally, to improve its pharmacokinetic profile, we also developed a liposomal formulation of RDD-142 by a simple, rapid, organic solvent-free procedure that allows the formation of stable vesicles in the nanoscale range. Since the formulation is intended for parenteral administration, the liposomes were prepared with PEGylated phospholipids to prolong circulation time in the bloodstream. Finally, we compared the cytostatic effects of free and liposomal RDD-142.

Overall, this study aims to investigate the molecular effects of RDD-142 in hepatocellular carcinoma cells and to assess whether its PEGylated liposomal formulation can enhance its therapeutic performance, thereby providing a preliminary preclinical framework for repurposing antiretroviral agents against HCC.

## 2. Results

### 2.1. RDD-142 Negatively Influences Neoplastic Cell Proliferation

Preliminary data obtained with RDD-142 demonstrated its cytotoxic activity on different hepatocellular carcinoma (HCC) cell lines, with a slight effect also on health counterpart IHH (Immortalized Human Hepatocyte) cells. Moreover, we concurrently demonstrated that RDD-142 decreased p-AKT levels in HCC in a dose-dependent manner [27], suggesting an involvement in the inhibition of the AKT signaling pathway.

Since AKT activation influences the proliferation of cancer cells [28], we investigated deeply the biological effect of RDD-142 on hepatocarcinoma HepG2 cells using an alternative method for cell viability assessment. Specifically, the xCELLigence system allows for monitoring, in real-time and without labeling, the dynamic changes in cell proliferation and viability through cell index (CI) variations caused by a treatment [29].

The xCELLigence technology works by measuring electron flow transmitted between gold microelectrodes, fused to the bottom surface of a 16-well microtiter culture plate, in the presence of an electrically conductive solution such as culture medium. Adhering cells disrupt the interaction between the electrodes and the bulk solution and thus impede electron flow. This electric impedance (resistance to alternating current) is expressed as arbitrary units called the cell index. The CI magnitude depends on the cell-seeded number, cell morphology, and cell size, as well as on the strength of cell attachment to the sensor electrodes. As cells adhered to the electrodes and proliferated, electronic impedance increased, while arrest of growth or detachment due to cell damage decreased impedance compared to the experimental control.

**Figure 2:**
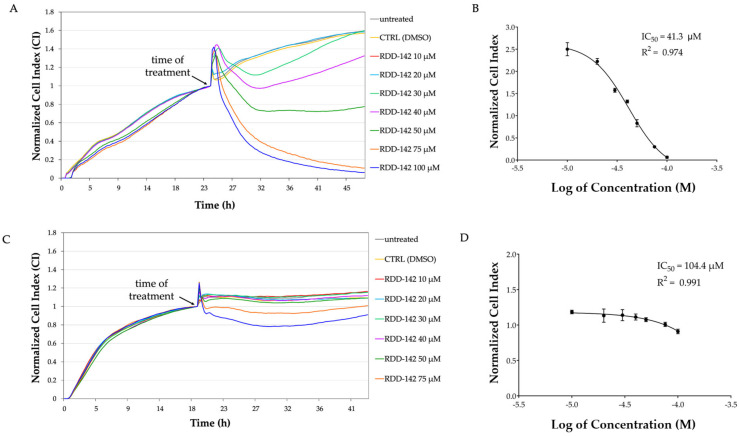
Effect of RDD-142 treatment on cell proliferation. Representative proliferation profile of HepG2 (**A**) and IHH (**C**) under 24 h of treatment with RDD-142 at different concentrations (10–100 µM range), assessed using the xCELLigence system. The cell index (CI) was normalized to the last cell index recorded before RDD-142 addition, measured in real time. Representative IC_50_ curves of RDD-142 in HepG2 (**B**) and IHH (**D**) cells calculated as means of normalized cell index ± SEM (*n* = 3). The normalized CI values, reported in the dose–response curve of RDD-142, were derived from the final measurements, taken after 24 h of treatment, at each tested inhibitor concentration.

The CI of HepG2 and IHH cells was registered in real time, before and after treatment with RDD-142, at a concentration range from 10 to 100 µM. As evident in Figure 2, CI values decreased as RDD-142 concentration increased, indicating an antiproliferative activity of this inhibitor more markedly on the neoplastic cell line (Figure 2A) than the non-tumorigenic line (Figure 2C), already at a concentration lower than the preliminary estimated IC_50_ of 67.96 µM [27]. RDD-142 caused a cell proliferation arrest in a dose-dependent manner, becoming statistically significant approximately at a concentration of 40 µM after 24 h from RDD-142 addition to the well, as demonstrated by the decrease in CI in RDD-142-treated cells compared to control cells (cells treated with DMSO). At concentrations higher than 50 µM, the CI decreased constantly, suggesting the detachment of cells from the bottom of the wells due to the pro-apoptotic activity of RDD-145 at around 70 µM. The IC_50_ value for HepG2 cells, determined by xCELLigence, was 41.3 µM (Figure 2B) after 24 h of treatment, lower than the value previously obtained by MTT [27]. Notably, RDD-142 treatment had a limited effect on IHH cell proliferation, for which the novel IC_50_, determined by xCELLigence experiment, was twice and a half higher than for neoplastic cells (Figure 2D).

The CI of treated cells was recorded for more than 24 h, but no further decrease of IC_50_ values was observed, suggesting that RDD-142 exerts its antiproliferative activity on neoplastic cells rapidly. For this reason, the successive experiments were performed treating HepG2 cells for 24 h with increasing concentrations of RDD-142 up to 42 µM, considered as the IC_50_ value at which the drug works as a cytostatic agent.

### 2.2. RDD-142 Attenuates Proliferation of Hepatocarcinoma Cells by Delaying Cell Cycle Progression

The preliminary xCELLigence data on cytostatic activity of RDD-142 were confirmed by performing the cell cycle analysis, with the cytometric analysis of propidium iodide (PI) on HepG2 and IHH cells after 24 h of treatment with RDD-142.

**Figure 3:**
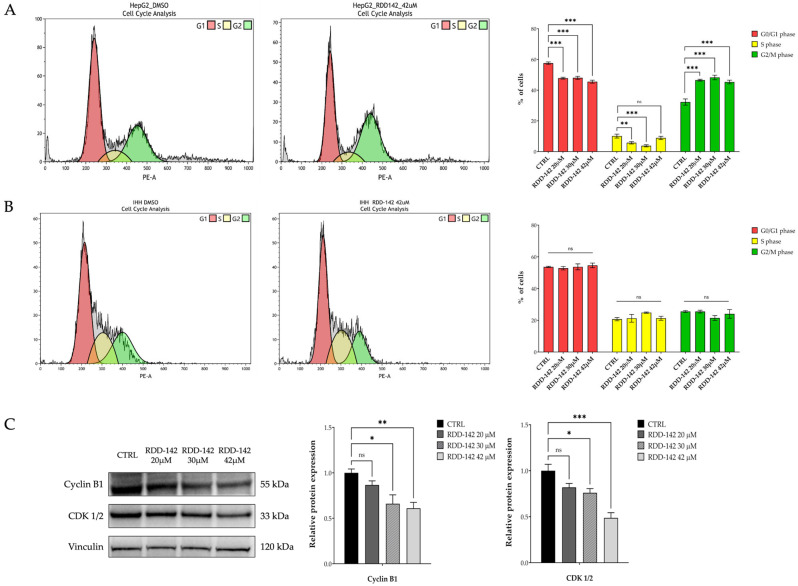
HepG2 accumulation in G2/M phase of the cell cycle after RDD-142 treatment. Cytofluorimetric analysis of the cell cycle of HepG2 (**A**) and IHH (**B**) cell lines after treatment with increasing concentrations of RDD-142 (20 µM, 30 µM, and 42 µM). Cell cycle histograms, on the left, show a single representative experiment of the proliferation profile of cells after 24 h of treatment with DMSO and RDD-142 at 42 µM. The bar graphs on the right represent the average of cells in all phases of the cell cycle, expressed as means ± SD of three independent experiments; ** *p* ≤ 0.01; *** *p* ≤ 0.001. Immunoblot analysis (**C**) for the evaluation of Cyclin B1 and CDK 1/2 expression levels, as markers of cell cycle progression, in HepG2 cells treated with increasing concentrations of RDD-142 (20 µM, 30 µM, and 42 µM) for 24 h. Densitometry analysis, on the right, was performed by normalizing with the respective vinculin and the values are expressed as means ± SD of three independent experiments; ns, not significant; * *p* ≤ 0.05; ** *p* ≤ 0.01; *** *p* ≤ 0.001.

The treatment of HepG2 cells with RDD-142 revealed a cell cycle slowing in G2/M phase (Figure 3A,B) already at a 20 mM concentration, compared to the DMSO control, with a significant accumulation of cells in this phase. This biological effect was also obtained at 30 and 42 mM of RDD-142, with almost 50% of cells blocked in G2/M at the concentration of 30 mM. At the same concentrations, IHH cells grew without any perturbation in distribution among the phases.

The immunoblotting analysis (Figure 3C) validated the FACS results, showing a statistically significant decrease in expression of CDK 1/2, a crucial kinase involved in cell cycle progression at G2/M transition, and Cyclin B1, a protein that regulates cell division, also binding to and activating cyclin-dependent kinase 1 (CDK1). The downregulation of these proteins started at the RDD-142 concentration of 20 µM, but it became statistically significant at 30 µM. Altogether, these data suggest that RDD-142 may induce a slight accumulation in G2/M phase due to a delay in cell cycle progression of the hepatocarcinoma cell line, at concentrations lower than its IC_50_ value as a cytotoxic agent [27]. In this way, healthy hepatocytes are preserved, allowing a reduction in side effects.

### 2.3. RDD-142 Induces Impairment of Cell Cycle Transit by Inactivation of ERK1/2 Signaling and Activation of Autophagy in HepG2 Cells

**Figure 4:**
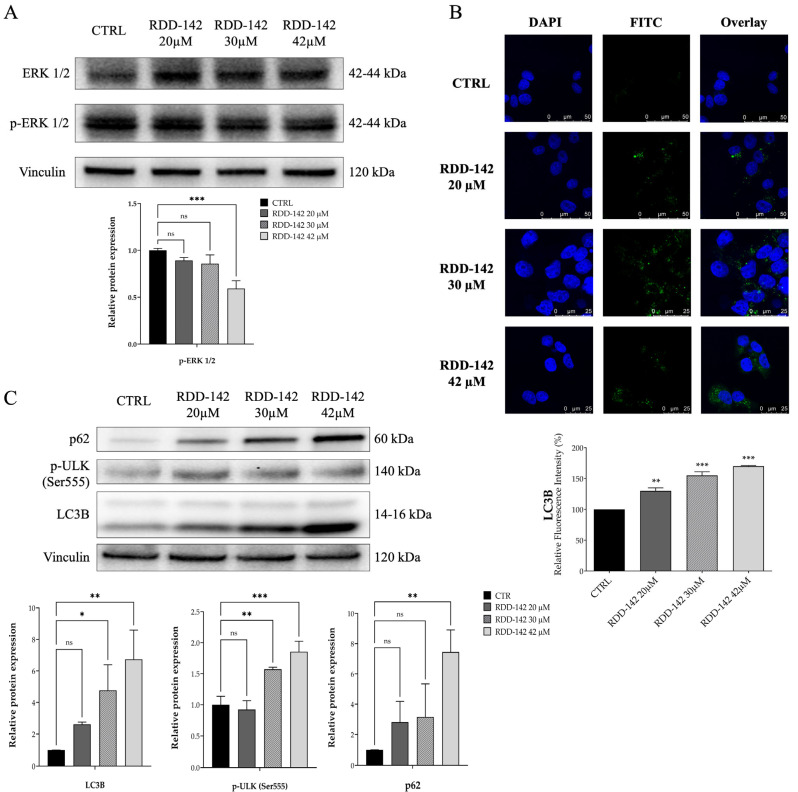
ERK1/2 attenuation and autophagy activation after RDD-142 treatment. (**A**) Protein expression level of ERK1/2 and its activation, evaluated by a Western blotting experiment after treatment with increasing concentrations of RDD-142 (20 µM, 30 µM, and 42 µM) for 24 h. Densitometry analysis for Western blotting experiments, below immunoblotting panels, was performed by normalizing with the respective vinculin, and the values are expressed as means ± SD of three independent experiments; ns, not significant; *** *p* ≤ 0.001. (**B**) LC3B expression evaluated by confocal microscopy after RDD-142 treatment. The bar graph, at the bottom, represents the percentage of LC3B fluorescence (means ± SD) of three acquisition fields; ** *p* ≤ 0.01; *** *p* ≤ 0.001. (**C**) Immunoblotting analysis for the determination of protein expression levels of autophagy after 24 h of treatment with RDD-142 at increased concentrations. Densitometry analysis for Western blotting experiments, below immunoblotting panels, was performed by normalizing with the respective vinculin, and the values are expressed as means ± SD of three independent experiments; ns, not significant; * *p* ≤ 0.05; ** *p* ≤ 0.01; *** *p* ≤ 0.001.

It is known that the Ras/Raf/MEK/ERK pathway is involved in tumor development and progression. In particular, ERK1/2 kinases promote cell cycle progression of both G1/S and G2/M phases in hepatocellular carcinoma and potentially inhibit apoptosis [26]. The dual specificity phosphatases (DUSPs) regulate the activity of ERK1/2 and other MAPKs through their dephosphorylation. The DUSP expression levels are regulated by ubiquitin-proteasome degradation; thus, proteasome inhibitors are able to affect ERK1/2 activation [30,31] by their phosphorylation levels. To determine the potential effect of RDD-142 on ERK1/2 activation, the immunoblotting of HepG2 cell lysate, after a treatment with increasing concentrations of RDD-142 for 24 h, was performed, evaluating the expression level of ERK1/2 and its phosphorylation at Thr202 and Thr204. As evident in Figure 4A, ERK1/2 phosphorylation at both threonine residues was gradually reduced with the increase in RDD-142 concentration, becoming statistically significant at the IC_50_ concentration. Instead, the total protein level of ERK1/2 remained constant, suggesting that RDD-142 controlled its activation rather than its expression.

ERK1/2 contribution to autophagy is dual and can either promote it or inhibit it, depending on the context of the cells. Generally, in nutrition deprivation and under stress stimuli, like ER-stress, ERK1/2 sustains autophagy by activating transcriptional factors, which regulate autophagy-related genes, and/or phosphorylates key proteins of autophagy involved in autophagy initiation [32,33]. The reduction in ERK1/2 phosphorylation suggested a suppression of the autophagic pathway, even if it was found by our group that RDD-142 promoted autophagy by controlling p-AKT level [27]. Because, in this study, the HepG2 cells were treated with RDD-142 concentrations reduced by 40% and 70% of its cytotoxic concentration, it was important to verify whether the biological effect of RDD-142 was also retained under these conditions of treatment. Thus, experiments of immunoblotting and confocal microscopy were carried out to evaluate autophagy activation by treating hepatocarcinoma cells with concentrations of RDD-142 (20 µM, 30 µM, and 42 µM) below its cytotoxic IC_50_.

The microscopy images (Figure 4B) highlighted that lower concentrations of RDD-142 had no significant effect on the formation of autophagosomes, but a statistically significant increase in fluorescence was evident already at the concentration of 20 µM, reaching over 50% of fluorescence increase at 42 µM. Western blotting also evidenced an increment of expression levels of p-ULK (Ser555), the initiating factor of autophagy, and p62, a classical receptor of autophagy (Figure 3C), confirming the activation of the autophagy pathway under RDD-142 treatment, despite ERK1/2 inhibition.

### 2.4. RDD-142 Inhibits Proteasome Activity

We previously demonstrated that RDD-142 induced an accumulation of ubiquitinated proteins, suggesting that the proteasome might be its molecular target. In silico molecular docking analysis supported the plausibility of this hypothesis, showing that RDD-142 preferentially binds to the β1–β5 and β6–β7 interfaces with high affinity, but not to the β2 catalytic site [27].

**Figure 5:**
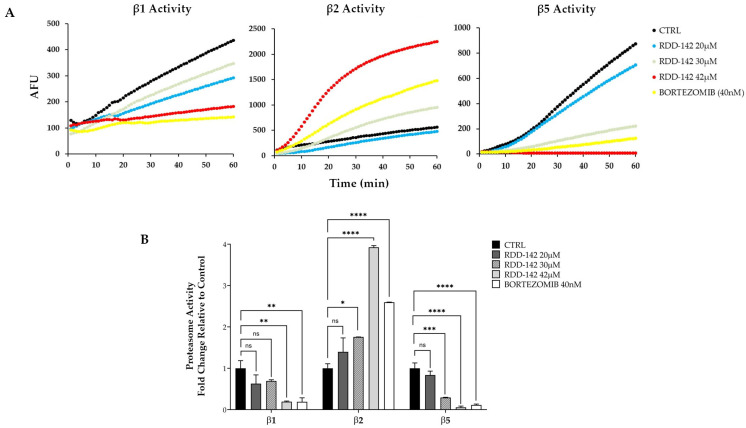
RDD-142 inhibits proteasome activity. (**A**) Representative kinetics of proteasome subunits β1 (caspase-like), β2 (trypsin-like), and β5 (chymotrypsin-like) activities in HepG2 cell lysates following treatment with RDD-142 (20, 30, and 42 µM), DMSO (CTRL), or Bortezomib (40 nM, positive control). Fluorescence was recorded at 1 min intervals and plotted as arbitrary fluorescence units (AFUs), corresponding to the raw intensity of the AMC signal released during the enzymatic reaction, versus time. (**B**) Quantification of proteasome activity expressed as the fold change relative to the control. Data represent means ± SEM of three independent experiments. Statistical significance: ns, not significant; * *p* < 0.05, ** *p* < 0.01, *** *p* < 0.001, **** *p* < 0.0001.

To further confirm these findings, we monitored the chymotrypsin-like, trypsin-like, and caspase-like protease activities associated with the proteasome complex in cell lysates from HepG2 cells treated with 20, 30, and 42 µM RDD-142, DMSO (negative control), or 40 nM Bortezomib (positive control). Figure 5A shows representative results from these experiments. Fluorescence was measured at 1 min intervals, and the data were plotted as arbitrary fluorescence units (AFUs), corresponding to the raw intensity of the AMC signal released during the enzymatic reaction, as a function of time.

The slope of the fluorescence curves, representing enzyme activity, was calculated and expressed as the fold change relative to the control (Figure 5B).

Following treatment with RDD-142, we observed a dose-dependent decrease in caspase activity (subunit β1, Z-LLE-AMC) and chymotrypsin-like activity (subunit β5, Suc-LLVY-AMC), while no inhibition of β2 (trypsin-like) activity was observed. This is consistent with the molecular docking results, suggesting that the lack of effect on β2 activity reflects the structural binding preferences of RDD-142 and supports its selective inhibition of β1 and β5 subunits.

### 2.5. RDD-142 Inhibits Proteasome Activity In Vitro

**Figure 6:**
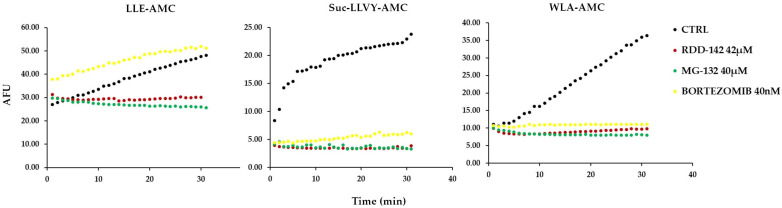
RDD-142 inhibits proteasome activity in vitro. Representative results showing the specific activity of the 20S proteasome after treatment with RDD-142 (42 µM), Bortezomib (40 nM), MG-132 (40 µM), or DMSO (vehicle control). Fluorescence was recorded at 1 min intervals and plotted as arbitrary fluorescence units (AFUs), corresponding to the raw intensity of the AMC signal released during the enzymatic reaction, versus time.

The 20S proteasome, a critical component of the ubiquitin–proteasome system involved in cellular protein regulation, is widely recognized as a key target of anticancer drugs [34,35,36]. Therefore, to determine whether RDD-142 could directly inhibit the proteasome, we measured 20S proteasome activity in vitro. The experiment was performed using RDD-142 at 42 µM (IC_50_ value), DMSO as a vehicle control, and either 40 µM MG-132 or 40 nM bortezomib as positive controls. As shown in Figure 6, MG-132 and bortezomib, known proteasome inhibitors, directly inhibited proteasome activities (purified proteasome provided by the kit), with MG-132 inhibiting both the β1 subunit (LLE-AMC) and the β5 subunit (Suc-LLVY-AMC, WLA-AMC), while bortezomib, as confirmed by the literature [37], only inhibited the β5 subunit. Regarding RDD-142, previous results suggested activity mainly on the β1 and β5 subunits, a finding also confirmed by this in vitro assay. However, as shown in Figure 6, a greater affinity of RDD-142 for the β5 subunit was observed. Therefore, it can be concluded that RDD-142 directly inhibits proteasome activity, with a specific effect on its chymotrypsin-like activity.

### 2.6. RDD-142 Formulation in PEG-Liposomes

Given the insolubility of RDD-142 in aqueous media, which results in reduced bioavailability and hampers its potential clinical application, the present study assessed its delivery in liposomes designed for parenteral administration. The liposomes are expected to increase RDD-142’s solubility, bioavailability, and cellular internalization, while being an aqueous-based formulation that can be safely administered to exert an antitumor activity. Key parameters influencing the functionality of RDD-142 PEGylated liposomes were studied. Several qualitative/quantitative liposome compositions were explored, and the formulation reported in Table 1 was found to be the best-performing one. In parallel to RDD-142 PEG-liposomes, empty PEG-liposomes and non-PEGylated liposomes were prepared to assess the effect of the drug’s incorporation and the PEGylation on the vesicles’ features.

The light scattering results, as reported in Table 1, showed that the empty PEG-liposomes were 91 nm in diameter, monodispersed (polydispersity index of 0.28), and negatively charged (ZP −25 mV). The incorporation of RDD142 decreased the vesicles’ mean diameter and reversed the zeta potential (Table 1).

The effect of the PEGylation was evaluated by comparing PEG-liposomes with non-PEGylated liposomes, both empty and loaded with RDD-142. As shown by the results reported in Table 2, the PEGylation induced an enlargement of the vesicles. Indeed, the non-PEGylated liposomes were significantly smaller than the corresponding PEG-liposomes. Moreover, the PEGylation modified the surface charge of the vesicles in a statistically significant manner, since empty liposomes showed zeta potential values close to neutrality. Again, the incorporation of RDD-142 had a marked impact on the vesicles, altering the arrangement of the phospholipids, which resulted in a reduction in size and an inversion of the zeta potential.

The entrapment efficiency of the RDD-142 PEG-liposomes, calculated after the purification process by dialysis, was high (89% ± 8.3).

**Figure 7:**
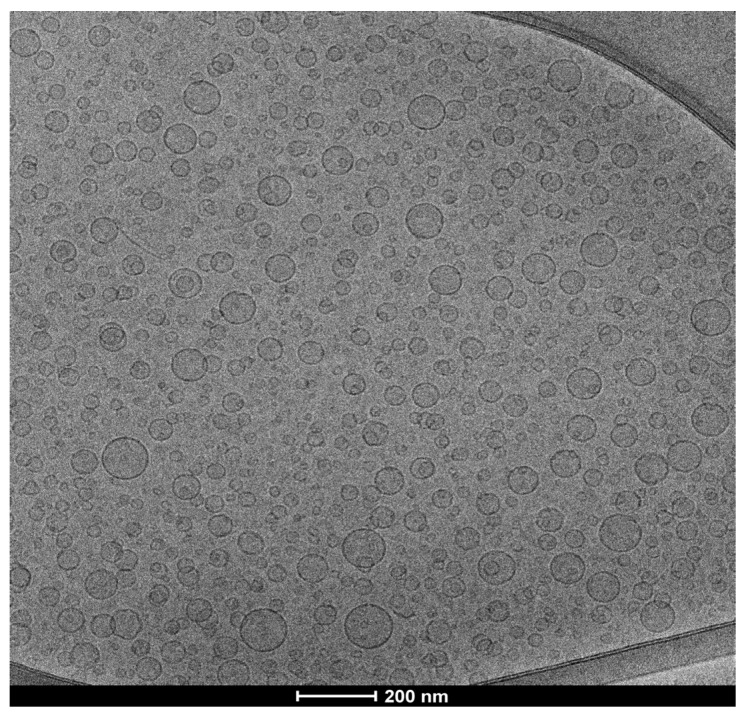
Cryo-TEM micrograph of RDD-142 PEG-liposomes. The image was recorded at a nominal magnification of 25,000×.

The observation of RDD-142 PEG-liposomes under cryo-TEM indicated the formation of spherical, unilamellar vesicles characterized by a diameter smaller than 100 nm (Figure 7), as also demonstrated by the light scattering data.

**Figure 8:**
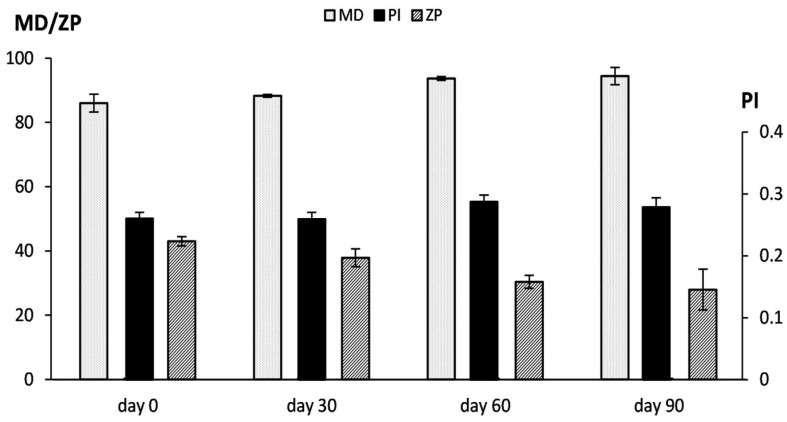
Colloidal stability of RDD-142 PEG-liposomes assessed by monitoring the mean diameter (MD), polydispersity index (PI), and zeta potential (ZP) for 90 days at 4 °C. Bars represent the mean values ± standard deviations (*n* = 4).

The stability of PEG-liposomes was evaluated during 3 months of storage. No macroscopic signs of physical alteration were detected, which was confirmed by the analysis of the mean diameter, polydispersity index, and zeta potential. The vesicles showed a tendency to increase in size and decrease in surface charge, but not in a statistically significant manner (Figure 8).

In addition, the stability of the PEG-liposomes was evaluated in a simulated biological fluid. Hanks’ solution is commonly used to simulate body fluids, as it contains inorganic salts and glucose that confer an osmotic pressure similar to that of intercellular fluids [38]. PEG-liposomes, both empty and loaded with RDD-142, were unaltered after dilution and incubation for 24 h with Hank’s solution (Table 2). The mean diameter and polydispersity index did not change in comparison with the initial values (t_0_), remaining around 85 nm and 0.2, respectively (Table 2). This indicates that their structure was maintained in spite of the osmotic stress caused by the high ion concentration in the Hank’s solution. The zeta potential values varied greatly, approaching neutrality already at t_0_ (Table 2), due to the ions adsorbed on the vesicles’ surface.

### 2.7. Liposomal RDD-142 as a Novel Formulation for a More Effective Antitumor Treatment

**Figure 9:**
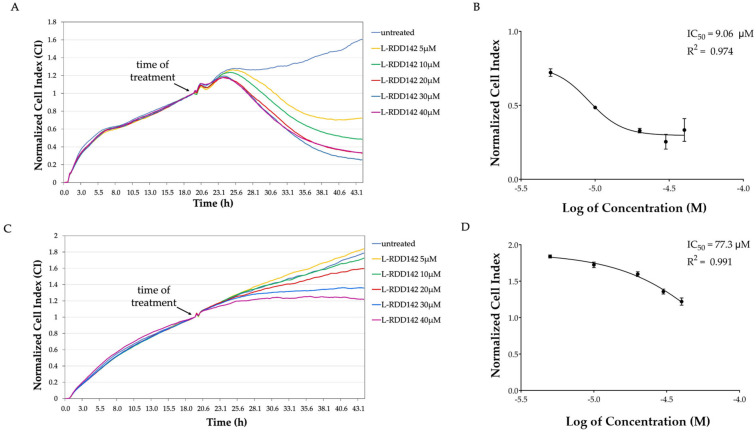
Effect of treatment with liposomal formulation of RDD-142 on HepG2 and IHH proliferation. Normalized cell index (CI) curves of HepG2 (**A**) and IHH (**C**) under 24 h of treatment with liposomal RDD-142 at different concentrations (5–40 µM range), assessed using the xCELLigence system. CI was normalized to the last cell index recorded before the addition of liposomal RDD-142, measured in real time. Representative IC_50_ curves of liposomal RDD142 in HepG2 (**B**) and IHH (**D**) cells calculated as means of normalized CI ± SEM (*n* = 3).

In a recent study, our group demonstrated that a liposomal formulation of a carbamate molecule, analogous to Darunavir, potentiated the drug’s cytotoxic activity without affecting its biological activity as a proteasome inhibitor and promoter of ER-stress [39]. For this reason, in this study, the efficacy of RDD-142 liposomal formulation on HepG2 neoplastic cells and the IHH healthy liver cell line was investigated. The cell viability was estimated by cytotoxic assay with xCELLigence, as already performed for the free RDD-142. The results confirmed a greater efficacy of the liposomal formulation, as demonstrated by the decrease in IC_50_ value for both tumor and healthy cells (Figure 9B,D). It is interesting to note that, for HepG2 cells, the IC_50_ of liposomal RDD-142 was more than four times lower than the IC_50_ of free RDD-142, while for IHH cells, the increase in antiproliferative effect of liposomal RDD-142 was minor. Observing the CI curves of HepG2 and IHH cells (Figure 9A,C), after treatment with liposomal RDD-142, the differences in the antiproliferative activity in tumor and healthy cells were evident. The CI of HepG2 cells decreased already at the lowest tested concentration (5 µM), while IHH cells were affected by treatment at 30 µM.

**Figure 10:**
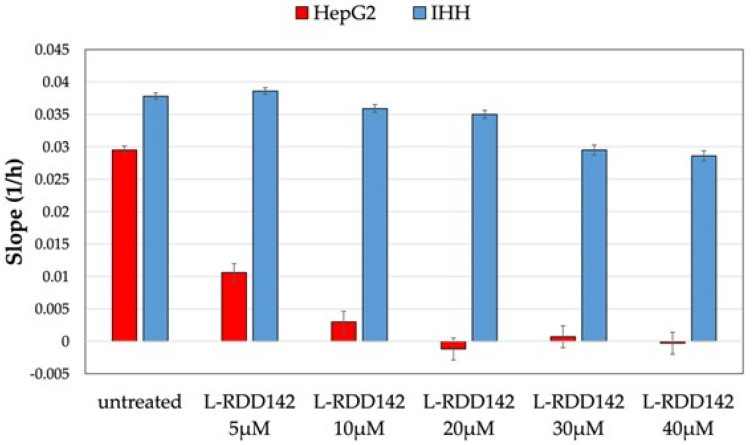
The slope of the cell index after treatment with liposomal RDD-142. The rate of proliferation of HepG2 and IHH cells at various concentrations of liposomal RDD-142 (5–40 µM range), as determined by analyzing the slope of cell index over 24 h.

Comparing the slope of HepG2 and IHH cells, obtained by xCELLigence experiments with liposomal RDD-142, the differences in antiproliferative activity on the two cellular models were evident (Figure 10). The slope represents the rate of change in CI over the duration of the experiment, or in a specific time window. This parameter, which is linked to the proliferation rate of cells, is useful for quantifying the behavior of cells on the chip because it depends on cell growth ability, attachment, and changes in shape. In this particular context, the slope graphs allowed us to speculate that the activity of liposomal formulation may be different in tumor and healthy cells. Indeed, for HepG2 cells, the slope became negative at a concentration of 40 µM, with a marked decrease in proliferation rate immediately at 5 µM. The negative value of the slope could indicate a detachment of cells from the chip surface, suggesting apoptotic death. Instead, IHH showed a comparable slope at a lower concentration of liposomal RDD-142, and a slight decrease in growth rate at 30 and 40 µM. These results underline the improvement in efficacy and selectivity via the nanoformulation of RDD-142 as an anticancer treatment.

### 2.8. Intracellular Intake of RDD-142

**Figure 11:**
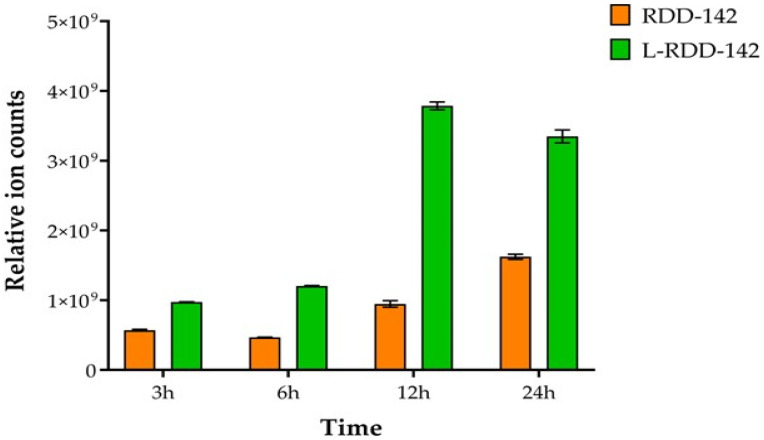
Intracellular intake of RDD-142. HepG2 cells were treated with 42 µM RDD-142, in solution or in PEG-liposomes (L-RDD-142) for 3, 6, 12, and 24 h. The amounts of RDD-142 accumulated in the cytoplasm are presented as areas of the chromatographic peak (means ± SD; *n* = 3).

LC–MS analyses were conducted to assess the cellular intake of RDD-142. HepG2 cells were incubated with 42 µM RDD-142 in either its free form (solution) or liposomal form for 24 h. The intake of RDD-142 increased in a time-dependent manner, particularly when delivered by PEG-liposomes. Notably, the PEG-liposomes enhanced the transport of RDD-142 across the cell membrane, as evidenced by a 2.5-fold higher concentration detected in the cytoplasm at 12 h and a 2-fold increase at 24 h (Figure 11). Importantly, no further increase in RDD-142 intake was observed after 24 h of exposure to the liposomal formulation. The intake of RDD-142 in its free form increased over time as well, although the levels were significantly lower compared to those observed with the liposomal formulation (Figure 11). This controlled and enhanced delivery, leading to a superior intracellular accumulation of RDD-142, likely contributes to the greater cytotoxicity observed when HepG2 cells were exposed to the nanoformulation, as compared to the free drug in solution.

## 3. Discussion

Drug repositioning is an innovative strategy to shorten drug discovery for novel cancer treatments. Since the step for safety assessment for use in humans has already been approved for the treatment of a specific disease, the traditional expensive and long process from basic science to the regulatory approval can be bypassed for a new use of existing drugs [5,6]. This approach has already been applied to antiviral protease inhibitors. Initially developed to target HIV proteases, ritonavir, saquinavir, indinavir, nelfinavir, and related compounds have been shown to directly interfere with tumor cell survival and proliferation, as well as angiogenesis, invasion, and inflammation [40]. In oncology, drug repositioning has gained special attention because it is more urgent to discover novel antitumor drugs in a short time. Furthermore, the high adaptability of cancer cells drives them easily toward drug resistance to standard treatments, making it essential to have compelling therapeutic strategies for patients.

HIV-protease inhibitors are in use for treatment of multiple myeloma and mantle cell lymphoma, and they have been studied in a variety of solid tumors, such as cervical [17,41], ovarian [14], bladder [12], and triple-negative breast cancer [42]. In spite of the incidence and high mortality of hepatocellular carcinoma (HCC), the antiproliferative effect of protease inhibitors has been poorly investigated. In a previous work, we demonstrated that RDD-142, a novel precursor of HIV-1 protease inhibitor Darunavir, may be a promising alternative therapy in HCC treatment. Nevertheless, it was necessary to deepen the biological characterization of this novel drug.

Major drawbacks of protease inhibitor use as cytotoxic agents in cancer include high dose requirements and severe side effects. It has been demonstrated that cytotoxic assay with the xCELLigence platform is comparable to classical MTS or MTT tests [29], but it is more sensitive to changes in adhesion, proliferation rate, and/or morphology of cells attached to xCELLigence E-plate support, after a drug treatment. Moreover, the xCELLigence technology allows the discrimination between cytostatic and cytotoxic activity of a screened compound based on real-time cell proliferation analysis. With the purpose of exploring the possibility of decreasing the concentration of RDD-142 treatment, preventing side effects on healthy cells, in this study, the anticancer activity evaluation of RDD-142 was performed using xCELLigence on both HCC and IHH cells. This approach warranted the estimation of a novel IC_50_ for RDD-142, lower than the previously found value, at which it exerts a cytostatic activity exclusively on cancer cells, preserving the healthy IHH cell line from any injury. A deep investigation of the biological activity of RDD-142 allowed us to demonstrate that, at lower concentrations, it modulates HepG2 cell proliferation through a delay in cell cycle progression, characterized by an extended duration in the G2/M phase. The control of cell cycle progression takes place via the inhibition of the ERK1/2 pathway. The MAPK signaling cascade regulates several cellular processes, like proliferation and survival, and it is frequently activated during ER stress and protease inhibition. This apparent discrepancy in our results may be clarified by taking into account that the downstream effect of PIs on signaling pathways seems pleiotropic and context-dependent. Indeed, the HIV-PI Nelfinavir has already demonstrated an anti-proliferative effect on tumor cells (medullary thyroid cancer, multiple myeloma, and breast cancer) by decreasing ERK phosphorylation. On the other hand, no effect on the inactivation of the MAPK pathway was observed in NSCLC, ovarian, and pancreatic cancer cells under Nelfinavir treatment [11].

In vitro experiments of proteasome activity, which were performed to explore the molecular mechanism by which RDD-142 activates UPR response, corroborate the attenuation of the ERK1/2 signaling induced by RDD-142 treatment. Indeed, RDD-142 showed an inhibitor effect on the proteasome, particularly inhibiting the chymotrypsin-like subunit, confirming that it may exert control on the MAPK transduction pathway. Other groups have already demonstrated that proteasome inhibitors may regulate MAPK/ERK signaling with subsequent stimulation of autophagy and apoptosis [43,44]. Interestingly, further analysis of the downstream biological effect of RDD-142 at novel IC_50_ established that this proteasome inhibitor still retains its pro-autophagic activity, linked to its proteasome-inhibitor activity, despite the reduction in treatment concentration. LC3B protein accumulation was observed along with an increase in the phosphorylation level of ULK1 at Ser555, the key regulator of autophagosome, in a dose-dependent manner. Interestingly, an increase in p62 expression at higher concentrations of RDD-142 was also found. This protein is the link between autophagy and ubiquitin–proteasome machinery [45], which confirms the triggering role of proteasome inhibition toward autophagy activation. Moreover, p62 is strictly linked to the ERK1/2 pathway in a feedback loop [46], which could explain the apparent inconsistency of our results. If, on one hand, ERK1/2 activation may promote p62 up-regulation by controlling its expression [47], then, on the other hand, p62 may interact with ERK kinases, influencing their activation negatively by promoting dephosphorylation [48]. Thus, attenuation of ERK1/2 signaling by RDD-142 could be the downstream effect of autophagy activation, as well as the cell cycle delay induced at a concentration lower than its cytotoxic IC_50_.

In the continuous effort to reduce the dose of proteasome and protease inhibitors to prevent cytotoxic effects on healthy cells, nanoformulation is a promising strategy to improve the drug’s bioavailability and bioactivity. In particular, liposomes are valid nanocarriers for drugs due to their chemical/structural nature, much more similar to cellular membranes than other nanostructures. Hence, liposomal formulation of RDD-142 was tested on HepG2 and IHH cells, in terms of cellular intake and cytostatic efficacy. As expected, the intake of liposomal RDD-142 increased, compared to the free drug, and contextually also its cytotoxicity. Further evaluation of the maximal inhibitory effect of liposomal RDD-142 by real-time analysis of cell proliferation demonstrated that the nanoformulated inhibitor preserved its cytostatic effect, with greater selectivity to hepatocarcinoma cells. Indeed, liposomal RDD-142 showed a reduction of IC_50_ of approximately 78%, while IC_50_ decreased for IHH cells by about 25%. These findings open new scenarios for the use of a nanoformulation approach to reduce the dose of cytotoxic agents.

## 4. Materials and Methods

### 4.1. Cell Lines: Culture Conditions and Treatments

Human hepatocellular carcinoma cell line HepG2 (ATCC #HB-8065; Manassas, VA, USA) was cultured in DMEM (Dulbecco’s Modified Eagle Medium) supplemented with 10% (*v*/*v*) fetal bovine serum (FBS), 2 mM L-glutamine, and 10 U/mL penicillin–streptomycin antibiotics. The healthy control of experiments was the IHH (Immortalized Human Hepatocyte) cell line, kindly provided by Prof. C. Tiribelli (Liver Research Center, Italian Liver Foundation, Trieste, Italy), propagated in DMEM/F-12 1:1 (Dulbecco’s Modified Eagle Medium/Nutrient Mixture F-12) supplemented with 10% FBS, 2 mM L-glutamine, 100 U/mL penicillin–streptomycin antibiotics, 120 nM insulin from bovine pancreas, and 1 µM dexamethasone. Both cell lines were grown in a humidified incubator at 37 °C in the presence of 5% CO_2_, routinely monitored by microscopic observation of cell morphology. Unless otherwise specified, reagents were purchased from Thermo Fisher Scientific (Waltham, MA, USA). The aminohydroxy sulfonamide *N*-(2*R*,3*S*)-3-amino-2-hydroxy-4-phenylbutyl)-*N*-benzyl-4-methoxybenzenesulfonamid, RDD-142, was synthesized as previously reported [26].

Cells were treated with the synthetic RDD-142 and liposomal RDD-142 for 24 h at known concentrations for experimental purposes. RDD-142 was dissolved in dimethylsulfoxide (DMSO) as a 50.0 mM stock solution and stored at −20 °C until use. The liposomal RDD-142 was prepared at a 5.0 mM stock concentration. The dilutions for the treatments were freshly prepared in the cell culture medium at the concentrations requested for the experiments.

### 4.2. Real-Time Cell Proliferation Monitoring by xCELLigence System

Cell proliferation experiments were performed using the xCELLigence System Real-Time Cell Analyzer (RTCA instrument, ACEA Biosciences, San Diego, CA, USA), as described in previous works [49,50]. Both IHH and HepG2 cells were seeded at an optimal number of 2 × 10^4^ cells/well in the E-Plate 16 plate, and their proliferation was automatically monitored every 30 min for 48 h. After an overnight incubation to allow the adhesion on the bottom of plate wells, the cells were treated with RDD-142 in a 10–100 µM range, using DMSO as a control, and liposomal RDD-142 in a 5–40 µM range. The cell index (CI) was recorded up to 72 h after treatment. Data were analyzed using xCELLigence software (Version 2.0, ACEA Biosciences) and expressed as a mean  ±  SEM of the CI of replicates, normalized to the last CI recorded before the time of molecule addition.

### 4.3. Cell Cycle Analysis

Cell cycle analysis was performed as previously reported [50] on both HepG2 and IHH cell lines, seeded in a 6-well plate at an optimal number of 2 × 10^5^ cells/well. After adhesion, cells were treated with RDD-142 at 20, 30, and 42 µM concentrations for 24 h, before sample preparation for cell cycle analysis. The flow cytometric analysis was carried out with the DxFlex instrument (Beckman Coulter, Brea, CA, USA). A total of 1 × 10^4^ events were acquired and analyzed for cell cycle analysis. The cell cycle results were expressed as a percentage of cells in G0/G1, S, and G2/M phases (mean  ±  SD of three independent experiments), analyzing the cell cycle distribution using Kaluza Analysis 1.3 software (Beckman Coulter).

### 4.4. Immunoblot Analysis

Total cell lysates of HepG2 cells treated with increasing concentrations of RDD-142 compound (20 µM, 30 µM, and 42 µM) were obtained by homogenization of cell pellets for 30 min in ice in a cold lysis buffer, made of 20 mM Tris, pH 7.5 containing 300 mM sucrose, 60 mM KCl, 15 mM NaCl, 5% (*v*/*v*) glycerol, 2 mM EDTA, 1% (*v*/*v*) Triton X-100, 1 mM PMSF, 2 mg/mL aprotinin, 2 mg/mL leupetin, and 0.2% (*w*/*v*) deoxycholate, with 1X halt protease and phosphatase inhibitor cocktails (#78429 and #1861277, Thermo Fisher Scientific Waltham, MA, USA). Immunoblot analysis was performed as previously reported [51] on 40 μg of total proteins extracted from lysed cells. Polyvinylidene difluoride membranes were incubated at 4 °C overnight with the following primary antibodies, conveniently diluted in Tween-Phosphate-Buffered Saline (T-PBS) as reported in the datasheet: CDK1/2 (sc-53219, Santa Cruz Biotechnology, Dallas, TX, USA), Cyclin B1 (sc-166757, Santa Cruz Biotechnology), LC3B (#2775S, Cell Signaling Technology, Danvers, MA, USA), p-ULK1 (S555) (#6888S, Cell Signaling technology), p62 (#66184-1, Proteintech, Rosemont, IL, USA), ERK1/2 (#9102S, Cell Signaling Technology), p-MAPK (T202/Y204) (#9106, Cell Signaling Technology), and Vinculin (sc-73614, Santa Cruz Biotechnology).

After incubation with a correspondingly suited horseradish peroxidase-conjugated secondary antibody (1:2000; Bio-Rad, Hercules, CA, USA), signals were developed using the enhanced chemiluminescence kit (Clarity™ Western ECL Substrate, Bio-Rad Laboratories Inc.) and detected using the chemiluminescence system ChemiDoc Imaging System XRS + with ImageLab software version 6.1 (BioRad Laboratories GmbH, Hercules, CA, USA)Protein expression was quantified by densitometric analysis, using the Quantity One 4.5 software (BioRad Laboratories GmbH, Hercules, CA, USA) and normalized according to the expression of the housekeeping gene. Densitometric analysis was performed on almost three independent replicates, and data were expressed as a mean  ±  SD of relative protein expression.

### 4.5. Immunofluorescence and Confocal Microscopy Analysis

For immunofluorescence experiments, about 4 × 10^4^ cells were seeded into 8-well chamber slides (Cell Culture Slide I, SPL Life Sciences Co., Ltd., Pocheon-si, Gyeonggi-do, Republic of Korea) and cultured at 37 °C for adhesion. The day after, cells were treated with RDD-142 at different concentrations (20, 30, and 42 µM) for 24 h before staining. After incubation, cells were washed with PBS and fixed with 0.1 M PBS containing 4% (*w*/*v*) paraformaldehyde (Sigma-Aldrich, Saint Louis, MO, USA) for 15 min at room temperature (RT). Then, cells were permeabilized with 0.1% (*v*/*v*) and blocked with a blocking buffer (5% BSA [*w*/*v*] in PBS) for 30 min at RT before staining with primary antibodies. Finally, the chambers were incubated overnight at 4 °C with 5% BSA (in PBS) containing LC3B primary antibody (#2775S, Cell Signaling Technology), diluted 1:100 in 5% BSA. After washing with PBS, an incubation with Alexa-Fluor 488 goat anti-rabbit IgG secondary antibody (#A-11070, ThermoFisher Scientific) was performed, and 1:1000 in PBS with 5% BSA was carried out for 1 h at RT in the dark. Cell nuclei were counterstained with 4′,6-diamidino-2-phenylindole (DAPI, ThermoFisher Scientific) as follows: after three washes with PBS, the slides were mounted using ProLong™ Glass Antifade Mountant with NucBlue™ (ThermoFisher Scientific) for cell nuclei counterstaining. Images were acquired by using a Leica SP8 confocal microscope (Leica Microsystems GmbH, Wetzlar, Germany) with a 40× objective, exciting the stained cells with 488 nm laser light. The intensity of LC3B signals was expressed as a percentage and normalized with respect to the number of nuclei in three distinct areas of the chamber.

### 4.6. Proteasome Activity Assay

Proteasome activity was assessed using a fluorescence-based assay detecting the cleavage of small peptides conjugated to the fluorogenic reporter 7-amido-4-methylcoumarin (AMC) [52]. Briefly, HepG2 cells (approximately 3 × 10^7^ cells per sample) treated with 20, 30 and 42 µM RDD-142, DMSO (negative control) or 40 nM Bortezomib (positive control) were collected and resuspended in 500 uL of ATP/DTT lysis buffer (Tris-HCl 10 mM, ATP 5 mM, DTT 0.5 mM, 5 mM MgCl_2_, pH 8) and incubated on ice for 30 min. Subsequently, sonication was performed for 15 s, and the lysates were centrifuged at 500× *g* for 10 min at 4 °C. The resulting supernatant contained proteasomes, and protein concentrations were measured by the Bradford assay with bovine serum albumin used as a standard [53]. The fluorogenic substrates Succ-LLVY-AMC, BZ-FVR-AMC, and Z-LLE-AMC (Vinci-Biochem Srl, Vinci, FI, Italy) were used to measure trypsin-like, chymotrypsin-like, and proteasome caspase-like activity, respectively. The assays were conducted using 20 μg of proteasome and 50 μM fluorogenic substrates in a total volume of 200 μL of ATP/DTT lysis buffer enriched with 50 mM EDTA. The reactions were incubated for 60 min, and fluorescence data (excitation wavelength at 360 nm, emission wavelength at 465 nm) were collected every 1 min using the instrument Thermo Scientific™ Varioskan™ LUX multimode microplate reader. The data for each sample were plotted as arbitrary fluorescence units (AFUs) versus time, and the slope of a line fit to the data was obtained using an appropriate linear regression program.

### 4.7. Analysis of Proteasome Activities In Vitro

To determine whether RDD-142 could directly inhibit proteasome activity (specifically chymotrypsin-like and caspase-like activities), we used the 20S Proteasome Kit (South Bay Bio, San Josè, CA, USA) according to the manufacturer’s protocol. Briefly, RDD-142 at a concentration of 42 μM was added to the solution containing the purified 20S proteasome and the AMC substrate of choice (Suc-LLVY-AMC, LLE-AMC, or WLA-AMC); bortezomib (40 nM) and MG-123 (40 uM) were used in parallel as a positive control. Fluorescence was measured using Thermo Scientific™ Varioskan™ LUX multimode plate reader (Ex/Em = 345/445 nm). Data for each sample were plotted as arbitrary fluorescence units (AFUs) versus time, and the slope of a line fit to the data was obtained using an appropriate linear regression program.

### 4.8. Vesicle Preparation and Characterization

RDD-142 PEGylated-liposomes were produced by dispersing RDD-142, Phospholipon90G (P90G, >94% phosphatidylcholine; Lipoid GmbH, Ludwigshafen, Germany), 1,2-Dipalmitoyl-sn-glycero-3-phosphocholine (DPPC; Lipoid Gmbh), and [N-(Carbonyl-methoxypolyethylene glycol-5000)-1,2-dipalmitoyl-sn-glycero-3-phosphoethanolamine, sodium salt] (MPEG-5000-DPPE; Lipoid GmbH) in water (Table 3) and sonicating this dispersion (6 cycles of 5 s on/2 s off + 8 cycles of 2 s on/2 s off) with a Soniprep 150 plus disintegrator (MSE Crowley, London, UK).

For comparative purposes, empty PEG-liposomes were prepared following the above procedure, but without the addition of RDD-142 (Table 3). Similarly, non-PEGylated liposomes, both empty and loaded with RDD-142, were prepared without the addition of MPEG-5000-DPPE (Table 3).

The formation and morphology of RDD-142 PEG-liposomes were examined via cryogenic transmission electron microscopy (cryo-TEM). Five µL of the vesicular dispersion was placed on a holey carbon grid, then blotted against filter paper. The resulting thin sample film was vitrified by plunging the grid into ethane, maintained at its melting point with liquid nitrogen using a Vitrobot (FEI Company, Eindhoven, The Netherlands). The vitreous film was transferred to a Tecnai F20 TEM (FEI Company) using a Gatan cryo-transfer (Gatan, Pleasanton, CA, USA), and the sample was observed in a low-dose mode. Images were acquired at 200 kV and at −170/−175 °C, using low-dose imaging conditions not exceeding 20 e^−^/Å2, with a 4096 × 4096 pixel CCD Eagle camera (FEI Company).

The average diameter, polydispersity index, and zeta potential of the vesicles were determined via dynamic and electrophoretic light scattering using a Zetasizer nano-ZS (Malvern Panalytical, Worcestershire, UK). The samples were diluted with water (1:100 *v*/*v*) and analyzed at 25 °C.

The vesicle dispersions were purified from the non-incorporated RDD-142 by dialysis. Each sample (1 mL) was loaded into 12–14 kDa MWCO Spectra/Por^®^ tubing (Spectrum Laboratories Inc., Breda, The Netherlands) and dialyzed against water (2 l) for 2 h to allow the removal of the non-incorporated drug. Both unpurified and purified vesicles were disrupted with methanol (1:100 *v*/*v*); RDD-142 content was determined by high-performance liquid chromatography (Alliance 2690, Waters, Milan, Italy), and the entrapment efficiency was calculated as the percentage of the amount of RDD-142 detected in purified vs. unpurified samples. RDD-142 was assayed using a fluorescence detector (λexc 240 nm and λem 285 nm), an X-Terra C18 column (3.5 µm, 4.6 × 100 mm, Waters), and a mobile phase consisting of acetonitrile, water, and acetic acid (99.5:0.48:0.02, *v*/*v*) delivered at a flow rate of 0.7 mL/min. LoQ was 10 pg.

### 4.9. Vesicle Stability During Storage and in Simulated Biological Fluid

The storage stability of PEG-liposomes was evaluated by monitoring vesicle mean diameter, polydispersity index, and zeta potential over three months at 4 °C. Furthermore, the stability in biological fluids was evaluated by incubation in Hank’s balanced salt solution (pH 7.4 ± 0.2) [54]. The mean diameter, polydispersity index, and zeta potential of PEG-liposomes were measured immediately after dilution (1:100 *v*:*v*) with the Hank’s solution, and after 24 h of incubation at 37 °C.

### 4.10. LC-MS/MS Analysis for the Evaluation of Cell Intake

HepG2 cells (2.5 × 10^5^ cells/well) were plated into 12-well plates and exposed to 42 µM RDD-142 (either in solution or formulated in PEG-liposomes) to assess the cellular intake of RDD-142. After 3, 6, 12, and 24 h, the cells were harvested. For the extraction of compound RDD-142, 500 µL of 40% methanol with 0.1% formic acid (*v*/*v*) was added to the cell pellets, followed by 1 min of sonication and 1 min without (10 min total) to lyse the cells. The resulting lysates were centrifuged for 5 min at 1500 rpm, and the supernatants were collected and concentrated with vacuum centrifugation [55]. The residues were then dissolved in methanol (200 µL) and analyzed by LC-MS using an Ultimate 3000 UPLC system coupled to a Q Exactive Orbitrap high-resolution mass spectrometry (Thermo Fisher Scientific, Milan, Italy). The analysis utilized a Luna reverse phase C18 column (150 × 2 mm, 3 µm, Phenomenex, Torrance, CA, USA) with a mobile phase containing water with 0.1% formic acid (phase A) and acetonitrile (phase B), applying a gradient of mobile phase B from 30% to 100% over 15 min. Full MS data were collected in positive ion mode using the following mass spectrometer parameters: capillary temperature of 320 °C, as well as sheath gas and auxiliary gas flow rates set to 35.0 and 15 arbitrary units, respectively, with a spray voltage of 3 kV. The precursor ion at 441.1843 *m*/*z*, representing the protonated form of RDD-142 [M + H]^+^, was used for quantification. Data analysis was performed using Xcalibur software version 2.2 (Thermo Fisher Scientific).

### 4.11. Statistical Analysis

Data are presented as means ± Standard Deviation (SD) or Standard Error of Measurement (SEM). Statistically significant differences (*p* < 0.05) were determined by one-way analysis of variance (ANOVA) followed by Dunnett’s post hoc test or by unpaired, two-tailed *t*-test, using Prism software v. 10.5.0 (GraphPad, Boston, MA, USA).

## 5. Conclusions

Even though an increasing number of studies support the efficacy of proteasome inhibitors as anticancer agents, their use comes with significant challenges, including potential toxicity and the risk of developing resistance to the drugs. One solution could be the development of delivery strategies that enhance the drug’s bioavailability, cellular intake, and specificity for neoplastic cells at a lower dose. Another purpose of researchers is exploring the combination of HIV-PIs with other therapies, such as chemotherapy, radiation, or targeted treatments, to enhance their effectiveness or improve the standard therapy efficacy in relapsed/refractory oncological patients, who have developed drug resistance or treatment toxicity.

In light of these considerations and our results, RDD-142 and its liposomal formulation may be evaluated as antiblastic agents, either as monotherapy or in combination therapy.

## Figures and Tables

**Table 1:** Characteristics of empty and RDD-142 PEG-liposomes and non-PEGylated liposomes: mean diameter (MD), polydispersity index (PI), and zeta potential (ZP). Each value represents the mean ± SD (*n* = 10). *, ** indicate values statistically different (*p* < 0.05 and *p* < 0.01, respectively) from empty PEG-liposomes; °, °° indicate values statistically different (*p* < 0.05 and *p* < 0.01, respectively) from empty liposomes; ^§§§^ indicate values statistically different (*p* < 0.001) from empty PEG-liposomes; ^##^, ^###^ indicate values statistically different (*p* < 0.01 and *p* < 0.001, respectively) from RDD-142 PEG-liposomes.

Formulation	MD nm ± SD	PI ± SD	ZP mV ± SD
Empty PEG-liposomes	91 ± 4.6	0.28 ± 0.03	−25 ± 4.1
RDD-142 PEG-liposomes	* 86 ± 3.2	0.26 ± 0.01	** +43 ± 2.5
Empty liposomes	^§§§^ 82 ± 1.5	0.29 ± 0.01	^§§§^ −6 ± 0.5
RDD-142 liposomes	^###^° 76 ± 3.2	^###^°° 0.32 ± 0.01	^##^°° +49 ± 2.5

**Table 2:** Mean diameter (MD), polydispersity index (PI), and zeta potential (ZP) of PEG-liposomes diluted and incubated with a simulated body fluid (Hank’s solution, pH 7.4) for 24 h, at 37 °C. The measurements were carried out immediately after dilution (t_0_) and after 24 h (t_24_) of incubation. Mean values ± SD are reported (*n* = 6).

Formulation	Time	MD (nm ± SD)	PI ± SD	ZP (mV ± SD)
Empty PEG-liposomes	t_0_	87 ± 0.9	0.21 ± 0.01	−2.4 ± 0.5
	t_24_	86 ± 0.7	0.21 ± 0.01	−2.2 ± 0.2
RDD-142 PEG-liposomes	t_0_	85 ± 2.2	0.22 ± 0.02	−0.2 ± 0.03
	t_24_	83 ± 2.5	0.20 ± 0.01	−0.2 ± 0.06

**Table 3:** Composition of the liposomal formulations.

Formulation	P90G	DPPC	MPEG-5000-DPPE	RDD142	H_2_O
Empty PEG-liposomes	40 mg	20 mg	2 mg	--	1 mL
RDD-142 PEG-liposomes	40 mg	20 mg	2 mg	5 mM	1 mL
Empty liposomes	40 mg	20 mg	--	--	1 mL
RDD-142 liposomes	40 mg	20 mg	--	5 mM	1 mL

## Data Availability

The original contributions presented in the study are included in the article. Further inquiries can be directed to the corresponding author.
